# Weakly bound water structure, bond valence saturation and water dynamics at the goethite (100) surface/aqueous interface: ab initio dynamical simulations

**DOI:** 10.1186/s12932-017-0040-5

**Published:** 2017-03-31

**Authors:** Ying Chen, Eric J. Bylaska, John H. Weare

**Affiliations:** 1 Department of Chemistry and Biochemistry, University of California, San Diego, La Jolla, CA 92093 USA; 2 William R. Wiley Environmental Molecular Sciences Laboratory, Pacific Northwest National Laboratory, Richland, WA 99354 USA

**Keywords:** Goethite, Goethite (100) surface, Fe-oxyhydroxide, Mineral water interface, Dissociative exchange, Ab initio molecular dynamics, DFT, Electronic structure, Bond valence theory, Water interaction with mineral surface, Condensed matter Grimme corrections

## Abstract

**Background:**

Many important geochemical and biogeochemical reactions occur in the mineral/formation water interface of the highly abundant mineral, goethite [α-Fe(OOH)]. Ab initio molecular dynamics (AIMD) simulations of the goethite α-FeOOH (100) surface and the structure, water bond formation and dynamics of water molecules in the mineral/aqueous interface are presented. Several exchange correlation functionals were employed (PBE96, PBE96 + Grimme, and PBE0) in the simulations of a (3 × 2) goethite surface with 65 absorbed water molecules in a 3D-periodic supercell (a = 30 Å, FeOOH slab ~12 Å thick, solvation layer ~18 Å thick).

**Results:**

The lowest energy goethite (100) surface termination model was determined to have an exposed surface Fe^3+^ that was loosely capped by a water molecule and a shared hydroxide with a neighboring surface Fe^3+^. The water molecules capping surface Fe^3+^ ions were found to be loosely bound at all DFT levels with and without Grimme corrections, indicative that each surface Fe^3+^ was coordinated with only five neighbors. These long bonds were supported by bond valence theory calculations, which showed that the bond valence of the surface Fe^3+^ was saturated and surface has a neutral charge. The polarization of the water layer adjacent to the surface was found to be small and affected only the nearest water. Analysis by density difference plots and localized Boys orbitals identified three types of water molecules: those loosely bound to the surface Fe^3+^, those hydrogen bonded to the surface hydroxyl, and bulk water with tetrahedral coordination. Boys orbital analysis showed that the spin down lone pair orbital of the weakly absorbed water interact more strongly with the spin up Fe^3+^ ion. These weakly bound surface water molecules were found to rapidly exchange with the second water layer (~0.025 exchanges/ps) using a dissociative mechanism.

**Conclusions:**

Water molecules adjacent to the surface were found to only weakly interact with the surface and as a result were readily able to exchange with the bulk water. To account for the large surface Fe–OH_2_ distances in the DFT calculations it was proposed that the surface Fe^3+^ atoms, which already have their bond valence fully satisfied with only five neighbors, are under-coordinated with respect to the bulk coordination.

**Graphical abstract:**
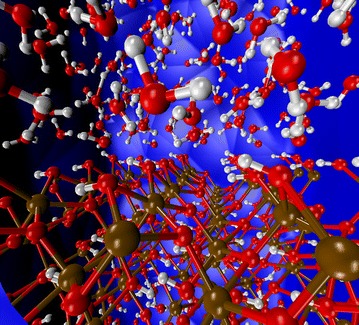
All first principle calculations, at all practically achievable levels, for the goethite 100 aqueous interface support a long bond and weak interaction between the exposed surface Fe^3+^ and water molecules capping the surface. This result is supported by bond valence theory calculations and is indicative that each surface Fe^3+^ is coordinated with only 5 neighbors.

## Background

As the thermodynamically most stable Fe-oxyhydroxide, goethite(α-FeOOH) occurs widely in natural environments [1–3] and is the dominant reactive mineral in lake and marine sediments [4]. It is found in weathering products, primary hydrothermal minerals, acid mine drainage precipitates, bog and marine environments [3–5] and has been observed in abundance on Mars [6]. The surface reactivity of the goethite-water interface has been extensively studied and is known to absorb a large number of reactive species including protons [7, 8], chromate [9], carboxylates [10], carbonate [11], arsenic [12, 13], and a host of soluble uranium [14, 15] and plutonium species [16]. To support interpretation of these processes we report here results of electronic structure simulations of the structure, reactivity and dynamics in the surface/aqueous liquid interface of this mineral.

**Fig. 1:**
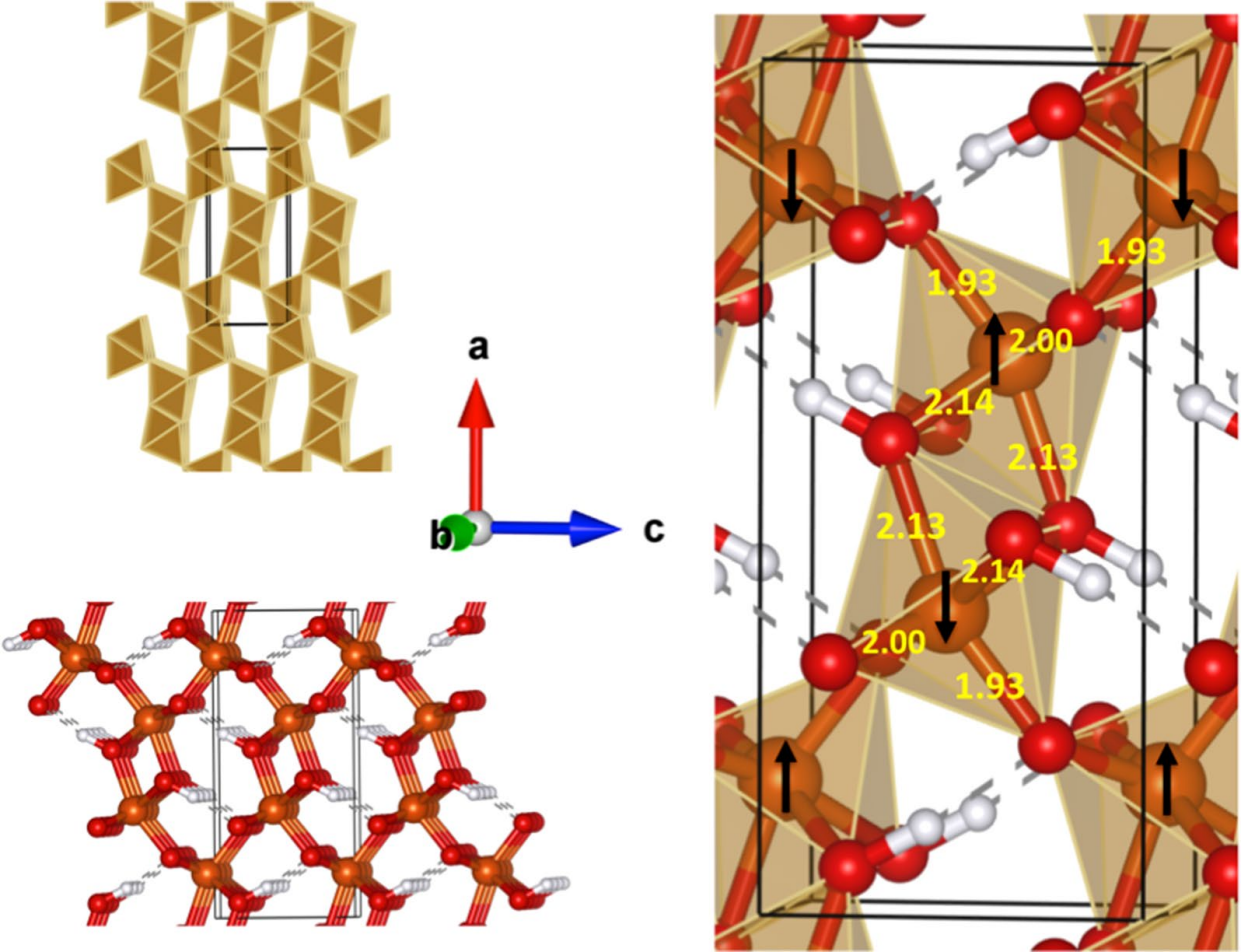
The crystal structure of bulk goethite (space group *No. 62*, *Pnma*) is shown in polyhedral, ball and stick, and combined forms. Note the a-axis is aligned vertically, the b-axis is aligned out of the page, and the c-axis is aligned horizontally. The bond distances (Å) in the combined form are results from this study for DFT PBE96 calculations. The *black arrows* represent the spin direction of the Fe atoms obtained from DFT PBE96 calculations

The goethite crystal structure has an orthorhombic dipyramidal class symmetry (space group *No. 62*, *Pnma* in the Hermann-Mauguin notation [17–19]) as shown in Fig. 1. Rather than the *Pnma* unit cell, it is common to use the *Pbmn* unit cell (see Rustad et al. [20] and Randall et al. [21]) with alternative axes specified by the following change of axes *(Pnma* → *Pbnm)*, *x* → *y*, *y* → *z, and z* → *x*. The crystal structure has a perfect cleavage on the (100) ((010) *Pbnm*) plane. This plane (more clearly illustrated below) is not the main crystal growth surface of this mineral. However it is a common surface and is easily cleaved to provide a well-structured surface for spectroscopic studies [22, 23].

Several molecular modeling studies of goethite and the goethite-water interfaces have been reported in the literature [24–28]. Most of these studies have used classical molecular dynamics (MD) to predict the structure and hydration behavior of these interfaces [24–33]. Multisite complexion models (i.e. MUSIC model [34, 35]) have also been used to predict their proton affinity. In addition to molecular dynamics studies, electronic structure calculations, i.e. DFT calculations with no dynamics, have been used to determine the structure and energetics of the goethite surfaces [2, 12, 21, 36], including those with absorbed water [2, 37]. The most thorough of these have been reported by Kubicki et al. [2]. They performed optimization studies for a single water absorbed on the goethite (100) surface termination at the DFT + U [38] level. In their calculations, the bond length between the first layer of water and the exposed surface Fe^3+^ atom were found to be very long (>2.3 Å); the adjacent water only weakly interacting with the surface Fe^3+^. This suggests that the exposed surface Fe^3+^ are fivefold coordinated rather than sixfold coordinated as in the bulk [39]. This under-coordination disagrees with prior MD simulations that reported a surface Fe^3+^–OH_2_ distance of 2.13 Å for the isolated surface (2.21 Å microsolvated) [27] and is does not support the accepted approach for generating surface models for oxides in which the metal cations retain the coordination of the bulk. De Leeuw and Cooper in prior work suggested such an under-coordination was possible for this surface even though their MD results predicted Fe^3+^–OH_2_ distances that were only slightly longer than bulk Fe–OH distances [27].

The results of Kubicki et al. also disagree with recent Crystal Truncation Rod (CTR) studies of the solvated goethite (100) surface by Ghose et al. [22] who obtained a distance of 2.152 Å from the analysis of their CTR intensities. This distance is slightly longer than the Fe–OH distances (~2.09 Å experiment, ~2.13 Å DFT) seen for the bulk (see Fig. 1). However, there is some uncertainty in the interpretation of the CTR spectra, because it was fit to a distribution of hypothesized structural models. In their paper, Kubicki et al. [2] rationalized the discrepancies of DFT from prior molecular dynamics results and the CTR experiment by pointing out that the bond length was very sensitive the solvation and additional layers of water molecules were needed to properly model the bulk goethite (100) water interface. Because of the computational cost they were not able to carry out these larger DFT simulations.

For weakly bound systems it is difficult to identify structures in a fluid that reliably reflect the average structure of the fluctuating system for use in direct optimization methods. While dynamics are available from MD, it can be quite challenging to accurately capture the strong polarization and other chemical interactions of the surrounding water molecules near complex transition-metal oxide surfaces [27, 32] using parameterized force fields. Ab initio molecular dynamics methods (AIMD) implement the calculation of forces on the fly from an approximate solution to the electronic Schrödinger equation (Density Functional Theory, DFT [38, 40, 41]). Chemical changes such as polarization, bond breaking and formation, etc. are straightforwardly incorporated in this approach, but at the cost of a much more expensive time step than MD. In this article we report results using AIMD simulations to predict the structural, bonding and dynamical properties of the solvated (~6 water layers) goethite (100) surface.

## Methods

All DFT [38] geometry optimizations and Car–Parrinello simulations [40, 41] in this study were performed with the plane-wave NWPW module [42] contained in the NWChem software package [43]. The DFT PBE96 [44] exchange correlation function was used for the majority of these calculations, however, the PBE96 + Grimme2 [44–46], and the hybrid [38] PBE0 [47] exchange correlation potentials were also used in the analysis. A recent review has proposed that Grimme corrections may be important in describing bulk water systems [46]. However, our careful comparisons of DFT results for Fe^3+^ and other first row transition metal aqua ions with experimental EXAFS data found that the effects of these additional corrections were quite small for solvated ions [48]. The interactions between valence electrons and the atom centers were approximated using generalized norm-conserving Hamann pseudopotentials [49, 50] for O, and H and a norm-conserving Troullier–Martin pseudopotential [51], which contained 4*s*, 4*p*, and 3*d* projectors and a semi-core correction was used for Fe. The pseudopotentials were modified into a separable form as suggested by Kleinman and Bylander [52]. For gradient corrected calculations, the NWPW module automatically generates pseudopotentials using the specified exchange correlation functional. The original pseudopotential parameterization suggested by Hamann were slightly softened by increasing the core raii: H: r_cs_ = 0.8 a.u., r_cp_ = 0.8 a.u.; O: r_cs_ = 0.7 a.u., r_cp_ = 0.7 a.u., and r_cd_ = 0.7 a.u. The radial cutoffs for the Troullier–Martin pseudopotential for Fe were r_cs_ = 1.24 a.u., r_cp_ = 1.24 a.u., r_cd_ = 1.23 a.u., and the s-channel pseudopotentials was chosen for the local potential in the Kleinman and Bylander expansion. Since this is a spin ordered system, unrestricted DFT calculations were performed. The electronic wavefunctions were expanded using a plane-wave basis with periodic boundary conditions at the Γ-point with a wavefunction cutoff energy of 100 Ry and a density cutoff energy of 200 Ry.

To establish the accuracy of the DFT PBE96 approach used in this manuscript, we evaluated its accuracy by calculating the bulk structural properties of the perfect goethite crystal. The orthorhombic unit cell contains 4 Fe atoms, 8 Oxygen atoms and 4 Hydrogen atoms (see Fig. 1). The lattice parameters were optimized using a 1 × 3 × 2 supercell Γ-point calculation. Relaxing the unit cell gave lattice parameters of 10.067, 9.155, and 9.204 Å (*a* = 10.067 Å, *b* = 3.0517 Å, *c* = 4.6020 Å), which are within 1.5% of experimental results [22, 53, 54]. These results are slightly better than found by Rosso and Rustad [55] in their DFT calculations, but not in as good agreement as those reported by Kubicki et al. [2], which were within 0.5% of experimental results [2] for lattice constants.

Goethite is antiferromagnetic at standard temperature (298.15 K) and pressure (1 atm). Three different spin orderings of the magnetic Fe atoms were calculated ([++−], [+−+], and [+−+−] along the *a* axis). A spin-penalty scheme was used to initialize the antiferromagnetic configurations [54]. The [+−+−] spin configuration shown in Fig. 1 was found to have the lowest energy (see Ying et al. [54]), which is in agreement with the prior calculations of Kubicki et al. [2]. These calculations predict a Fe site local spin S = 3.5/2 compared S = 3.8/2 seen in experiment [56].

To simulate the goethite (100) (or (010) Pbnm) surface a 30.0 Å × 9.155 Å × 9.204 Å periodic unit cell was used for all surface calculations in this study. This cell contains a 3 × 2 (100) surface slab with approximately 10 Å between slabs. The goethite surface slab contained 24 Fe, 48 O and 60 H atoms, and the thickness of the slab was 9.4 Å (distance in a direction between two oxygen atoms on surface). The slab has a relatively small number of 4 Fe layers, however, the expansion between the layers from the bulk values was observed to only be a couple of percent in these studies.

AIMD simulations were performed using open shell DFT. The system was propagated in time using Car–Parrinello Molecular dynamics (CPMD) scheme [40]. A plane wave basis and Γ point sampling were used to expand DFT wavefunctions. Simulations were carried out using both the PBE96 exchange correlation potential and PBE96 plus the Grimme2 correction for dispersion to check the importance of dispersion corrections for the water–water interactions [46]. The same pseudopotential and cutoff energies used in plane-wave optimization part were used in the dynamic AIMD simulations. Equation of motions in CPMD were integrated using position Verlet algorithm, with a time step 0.12 fs and fictitious orbital mass 600.0 au. All hydrogen atoms were replaced by deuterium to facilitate the integration. The simulation was carried out in a constant temperature canonical ensemble (300 K) using Nose–Hoover thermostats [57–59] to control the temperatures of the ions and the 1-electron orbitals.

The simulation of the solvated interface included 65 waters between the slabs (including 12 water molecules from the goethite surface). The water density in between the slabs was near 1.0 g/cm^3^. The relatively small thickness between the slabs results in a nano-confined water layer that will not formally capture several important properties of bulk water (e.g. diffusion, dielectric relaxation response), however the water layer is expected to be large enough to describe the structure of the water-surface interface. This large simulation contained 1024 valence electrons (512 spin up electrons and 512 spin down electrons). To initiate the simulation, the 53 water molecules in between the slabs were pointed away from hydrated goethite surfaces. An initial simulation time of at 1.5 ps was performed to equilibrate the system after which trajectory snapshots were collected.

## Results and discussion

### Termination of (100) surface without interface water layers

**Fig. 2:**
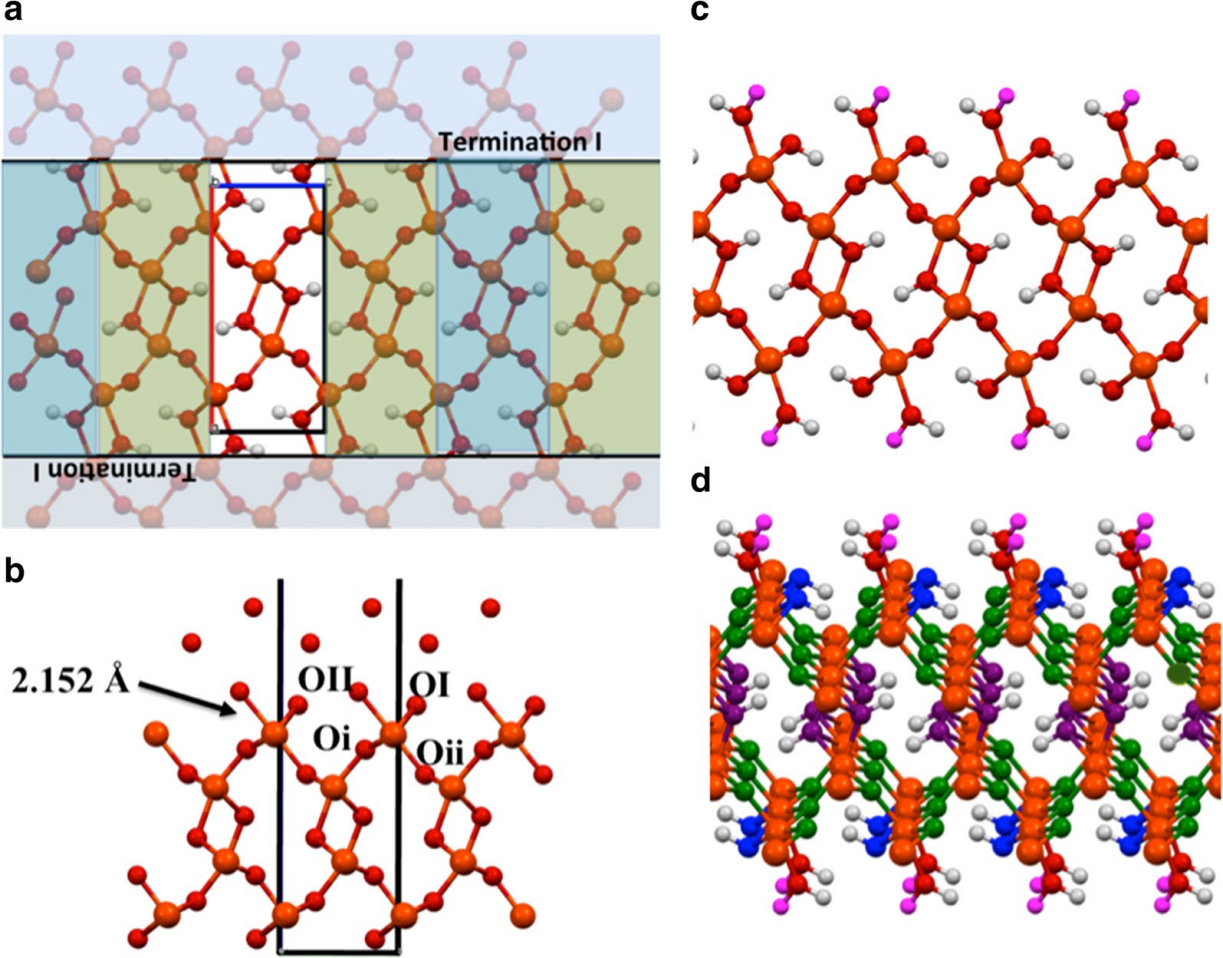
**a** Termination planes used to generate the Surface I slab. **b** Structural model for the solvated goethite (100) surface obtained from CTR experiments of Ghose et al. [22], which corresponds to surface I slab. Labels: OI—surface hydroxyl, OII surface water, Oi and Oii—bulk O^2−^ bonded to surface Fe. **c** The surface I slab obtained from the cleaving at the Termination I planes. The magenta colored hydrogen atom was added to neutralize the surface cleavage. **c** The oxygen atoms in the surface I slab are *color coded* to show the coordination. *Purple*—hydroxide with three Fe neighbors, Green—O^2−^ with three Fe neighbors, *blue*—hydroxide with two Fe neighbors

**Fig. 3:**
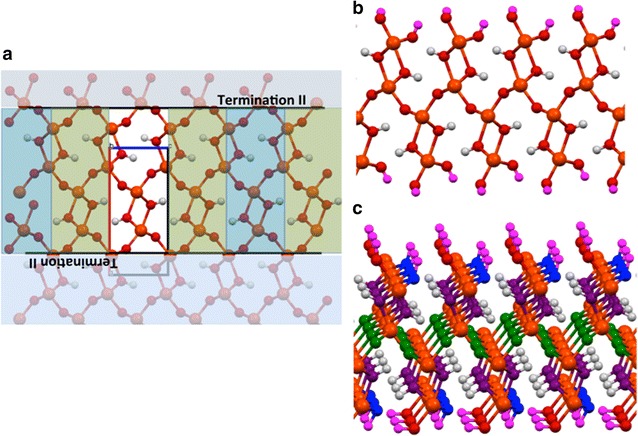
**a** Termination planes used to generate the surface II slab. **b** The isolated surface II slab obtained from cleaving at the termination II planes. The *magenta colored* hydrogen atom was added to neutralize the surface cleavage. **c** The oxygen atoms in the surface II slab are *color coded* to show the coordination. *purple*—hydroxide with three Fe neighbors, *green*—O^2−^ with three Fe neighbors, *blue*—hydroxide with two Fe neighbors

**Fig. 4:**
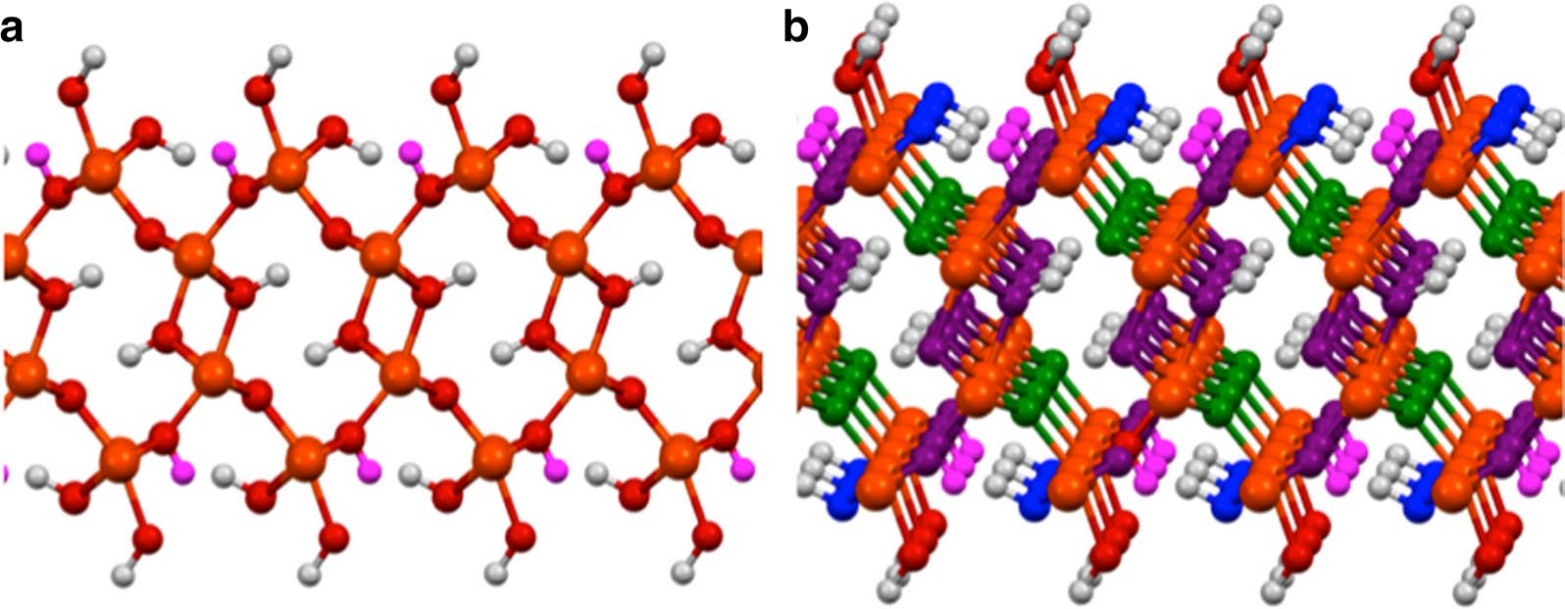
**a** The isolated surface ID slab obtained from cleaving at the termination I planes shown if Fig. 2a. The *magenta colored* hydrogen atom shows the alternative placement of the neutralizing hydrogen atom. **b** The oxygen atoms in the surface ID slab are *color coded* to show the coordination. Color coding: *purple*—hydroxide with three Fe neighbors, *green*—O^2−^ with three Fe neighbors, *blue*—hydroxide with two Fe neighbors

There are several possible surface cleavages for the goethite (100) surface in which the surface Fe^3+^ cations maintain the octahedral structure that is found in the bulk. Ghose et al. [22] in fitting their crystal truncation rod (CTR) data postulated four possible surface cleavages for this surface. We initially considered the two largest fractions of the surface cleavages obtained by Ghose et al. The first cleavage (surface I) shown in Fig. 2 has an OH^−^ layer on the surface. The second cleavage (surface II) shown in Fig. 3 has an O^2−^ layer on the surface. These two cleavages were labeled by Kubicki et al. [2] as the O^2−^ termination surface and the O^1−^ termination surface respectively (surface I → O^2−^ termination, surface II → O^1−^ termination). The unsaturated surface OH^−^ and O^2−^ anions in both these cleavages were protonated to neutralize the charge. The first cleavage has another possible protonation (surface ID) shown in Fig. 4, which we also considered. In this cleavage, also considered by de Leeuw and Cooper [27], the third highest oxygen in the cleaved structure is protonated instead of the top hydroxyl.

It is straightforward to compare the energies of the three surface models since they have the same number of atoms per unit cell (24 Fe atoms, 60 O atoms and 48 H atoms for the 3 × 2 surface cells). Energetic results for the three surface models at the PBE96 level are shown in upper part of Table 1. It was found that Surface I has the lowest energy, which is in agreement with the analysis of the CTR experiments [22] and the prior DFT + U calculations [2]. The next lowest energy structure is surface II, followed by surface ID.

**Table 1:** Total and relative energies from DFT PBE96 simulations for the vacuum surface models and solvated surfaces models of the (100) surface of goethite

Surface model	Molecular formula of supercell	Average total energy (a.u.)	Relative energy (kJ/mol)
Vacuum surface models			
3 × 2 surface I	Fe_24_O_60_H_48_	−1660.543130	0
3 × 2 surface II	Fe_24_O_60_H_48_	−1660.379184	430.6
3 × 2 surface ID	Fe_24_O_60_H_48_	−1660.328303	564.3
Solvated surface models			
3 × 2 surface I + 53H_2_O	Fe_24_O_113_H_154_	−2570.717514	0
3 × 2 surface II + 53H_2_O	Fe_24_O_113_H_154_	−2570.566486	397.7
3 × 2 surface ID + 53H_2_O	Fe_24_O_113_H_154_	−2570.498789	574.5

For the lowest energy surface, surface I, it was found that the water molecules capping each surface Fe, were very weakly bonded. Optimization of surface I resulted in a large Fe–OH_2_ bond distance of 2.45 Å. This is considerably larger than 2.15 Å distance in the model fitted from CTR data [22]. Kubicki et al. saw a similar result in their PBE96 + U calculations [2]. Further support of the weak interaction between water and surface Fe^3+^ was obtained from AIMD simulations of the isolated surface I (Fig. 2). These simulations performed at 300 K showed that the capping waters readily leave the surface to form a thin water layer. The other higher energy surface structures, surface II and surface ID, which were instead capped by hydroxide, had distances of 1.94 and 1.95 Å respectively. These distances are significantly smaller than the 2.15 Å distance predicted from the CTR analysis [22] and 2.13 Å distance from prior MD calculations [27].

Even though the large Fe–OH_2_ distances for the lowest energy surface (surface I) found in DFT calculations do not agree with prior MD calculations [8, 27, 28, 33] or with the structural model fit from CTR experiments [22], the large distances make chemical sense because (in a ionic lattice model) the negative charge due to the neighboring ions near the surface Fe^3+^ is −3. This makes the region near the surface Fe^3+^ effectively neutral, which in turn makes the Coulomb attraction to the dipole in the water molecule very small. To quantify this one can sum up the effective charge of the neighboring groups and add it to the +3 charge of the surface Fe^3+^ to determine its effective charging. Each surface Fe^3+^, without counting the surface water, is neighbors with 3 O^2−^ that are shared by 3 Fe^3+^ (shown as green spheres in Fig. 2c), and 2 OH^−^ that are shared by 2 Fe^3+^ (shown as blue spheres in Fig. 2c). This analysis shows that each surface Fe^3+^ is surrounded by a valence charge of 3(−2)/3 + 2(−1)/2 = −3 with only 5 neighbors. On the other hand, the bulk Fe^3+^ atoms are neighbors with 3 OH^−^ shared by 3 Fe^3+^ and 3 O^2−^ shared by 3 Fe^3+^, resulting in a valence charge of 3(−1)/3 + 3(−2)/3 = −3. This simple analysis clearly shows that the long surface Fe–OH_2_ distance can be rationalized by the fact that the surface Fe^3+^ atoms are already fully charge balanced (i.e. valence saturated) with only 5 neighbors.

A theoretical approach that formalizes the charge balance arguments above is bond valence theory [39, 60–62]. This theory, originally developed by Pauling [63], is widely used to predict bond lengths in minerals. The basic supposition of this theory is that the bond strengths around a cation (or anion) are set equal to the valence of the cation (anion) divided by its coordination number (assuming the same ligands). For example, each bond about an octahedral Fe^3+^ has a bond strength of 3/6 or ½. Similarly the bond strengths of the O–H bonds in a water molecule would be 2/2 or 1, since the oxygen atom has an oxidation state of −2 or in terms of valence is 2. The theory can be extended to predict bond lengths by further assuming that the bond strengths are inversely related to the bond lengths with the following formula

$$v_{ij} = { \exp }\left( {\frac{{R_{ij}^{0} - R_{ij} }}{B}} \right)$$

where *v*_*ij*_ is the bond strength and $R_{ij}$ is the bond length between atoms *i* and *j,* $R_{ij}^{0}$ is the distance for a bond between atom *i* and *j* to have a bond strength equal to 1, and *B* is an empirical constant. Brown and Altermatt [39, 60] have determined $R_{ij}^{0}$ for each kind cation-anion in the Inorganic Crystal Structure Data Base (ICSD) [64] in which the anions are chemically identical. The fitted values of $R_{ij}^{0}$ for Fe^3+^–O^2−^ and H^+^–O^2−^ are 1.759 and 0.882 Å respectively. During their fitting they also found that *B* varies very little from structure to structure, and they suggested using a fixed value of *B* = 0.37 Å. Setting the valences of each atom, *V*_*i*_, equal to the sum of its bond strengths

$$V_{i} = \mathop \sum \limits_{j} v_{ij} \quad {\text{or}}\quad V_{i} = \mathop \sum \limits_{j} \exp \left( {\frac{{R_{ij}^{0} - R_{ij} }}{B}} \right)$$

generates a system of non-linear equations for the bonding network, which can be solved for all the bond distances in the structure In goethite, the valences for Fe^3+^, O^2−^ and H^+^ are 3, 2 and 1 respectively. There are several strategies for solving this system of non-linear equations for the bond distances. We chose to solve them by minimizing the following penalty function

$$\chi\,=\,\mathop \sum \limits_{i} \left( {V_{i} - \mathop \sum \limits_{{\begin{array}{*{20}c} {j } \\ {{ \ni } i,j\, form\,bonds} \\ \end{array} }} { \exp }\left( {\frac{{R_{ij}^{0} - R_{ij} }}{B}} \right)} \right)^{2}$$

with respect to $R_{ij}$. The results of these calculations for the 3 × 2 Surface I model are given in the second column of Table 2 and show that the surface Fe–OH_2_ distances are very long and essentially unbound.

**Table 2:** Fe–O bond distances for the surface I slab model of the (100) surface of goethite

Surface Fe–O distances	BV	PBE96	PBE96 + Grimme2	PBE0	Exp. CTR model
Fe–OH_2_ (Fe–OII)	Unbound	2.45	2.39	2.41	2.15
Fe–OH (Fe–OI)	2.02	2.10	2.11	2.09	2.09
Fe–O (Fe–Oi, Fe–Oii)	1.91, 1.91	2.01, 1.95	2.01, 1.94	2.01, 1.98	1.95, 1.97

1st layer Fe–O distances	BV	PBE96	PBE96 + Grimme2	PBE0	CTR model
Fe–OH	2.17, 2.17, 2.17	2.14, 2.14, 2.19	2.15, 2.15, 2.16	2.13, 2.13, 2.16	2.19, 2.19, 2.10
Fe–O	1.91, 1.91, 1.91	1.94, 2.01, 2.01	1.93, 2.00, 2.00	1.98, 2.00, 2.00	1.94, 1.93, 1.93

See Fig. 4c for definitions of OI, OII, Oi, and Oii oxygens

It is also possible that electronic structure effects missing from the PBE96 exchange correlation functional may be able to contract the large Fe–OH_2_ distances. It is well know that DFT has major problems predicting the electronic structure near the Fermi level (e.g. band gap) [54] for transition metal oxides and oxyhydroxides and other strongly correlated systems. These errors at the Fermi level can sometimes, but not always, produce anomalous structures, e.g. including exact exchange might effect the charge distribution between the metal and oxygen atoms and, therefore the bond formation of the water to the surface. To check for these possible effects, hybrid DFT PBE0 (presumed to be a better level of exchange correlation in DFT [38]) were carried out for the 3 × 2 Surface I model. In addition, DFT as mean field theory cannot correctly treat long-range dispersion forces and as a result dispersion forces can be underestimated. To estimate the effects of dispersion, PBE96 + Grimme2 [46] calculations were also carried out for the 3 × 2 Surface I model. These calculations, results given in Table 2, also lead to large Fe–OH_2_ distances (2.45 Å PBE96, 2.39 Å PBE96 + Grimme2, and 2.41 Å PBE0). This suggests that the prediction of large distances is not overly sensitive to the level of theory used in the electronic structure calculations. These calculations clearly show that there is a strong disagreement in the results for the Fe–OH_2_ distances using different theories. Electronic structure calculations and bond valence theory calculations predict large distances, and prior classical molecular dynamics potentials and the model fitted to CTR experiments [22] predicts distances that are just slightly larger (~2.15A Å) than the bulk distances for Fe–OH in goethite (~2.09 Å).

### AIMD simulations of goethite (100) water interface

To further check the relative energetics of the three terminations and resulting surfaces we added 53 H_2_O molecules between the slabs and ran AIMD simulations using the PBE96 exchange correlation functional for at least 10 ps. Each of three simulations was performed using a unit cell that contained a total of 24 Fe atoms, 113 O atoms and 154 H atoms (Fe_24_O_60_H_48_ + 53 H_2_O). The simulations were designed to have water densities in between the slabs near 1.0 g/cm^3^. The average potential energies, given in the lower part of Table 1, show that the solvated surfaces have nearly the same relative energetics as the vacuum terminated surfaces. Given that Surface I was found to have a significantly lower energy than the other two surface models in vacuo and with solvation, the rest of the manuscript only presents results for this surface.

### The structure of the surface I  +  water interface

**Fig. 5:**
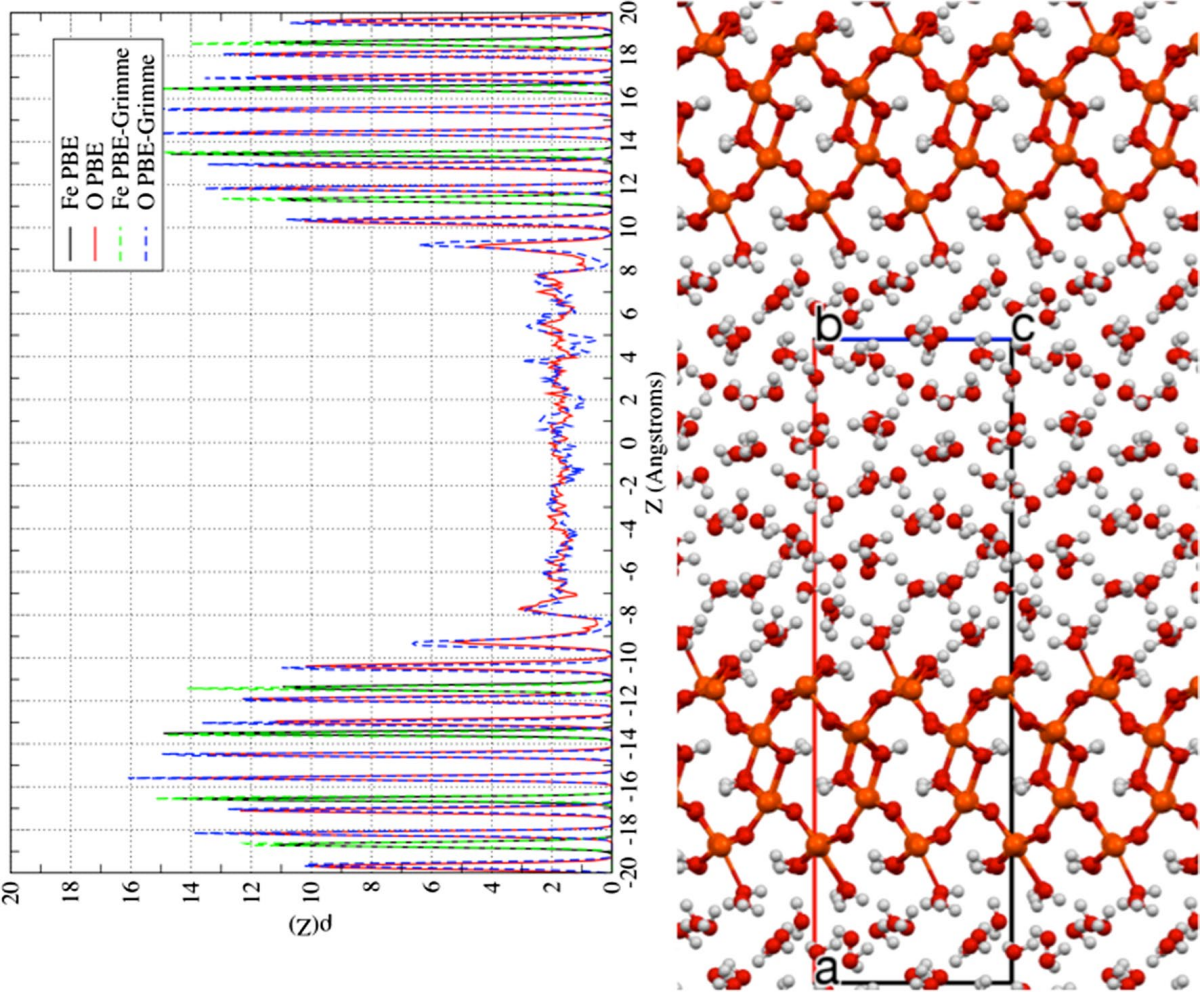
*Left* Laterally averaged oxygen atom and iron atom densities from PBE96 and PBE96 + Grimme2 AIMD simulations. *Right* Snapshot of PBE96 AIMD simulation aligned with densities

Simulations were carried out for this surface using both the PBE96 and PBE96 + Grimme2 exchange correlation functionals. The laterally averaged densities per supercell of the Fe and O atoms for the PBE96 and PBE96 + Grimme2 AIMD simulations are given in Fig. 5. Both the simulations produce well-defined and isolated peaks for the oxygen and iron densities for the goethite slabs, which are in the ranges between −20 to −9 Å, and 9 to 20 Å. In the optimized crystal at the PBE96 level the planes of iron atoms were separated by 2.08, 2.96, and 2.08 Å. Whereas in the slab, the distances between the planes were slightly expanded to be 2.11, 3.07, and 2.11 Å at the PBE96 level, and 2.13, 2.98, and 2.13 Å at the PBE96 + Grimme2 level. This results in an overall expansion of 2.3 and 1.6% between the outer planes of iron atoms at the PBE96 and PBE96 + Grimme2 levels respectively. This is in reasonable agreement given the relatively small slab models used in this study. Similarly the distances between the planes of oxygen atoms in the slab were slightly larger than found for the bulk. The oxygen planes were separated by 1.44, 1.07, 1.44, 1.08, 1.44, 1.07, 1.44 Å, for the bulk PBE96 crystal; 1.54, 1.07, 1.52, 1.08, 1.52, 1.07, and 1.54 Å for the slab at the PBE96 level; and 1.48, 1.09, 1.47, 1.09, 1.48, 1.09 and 1.48 Å for the slab at the PBE96 + Grimme2 level.

The first outside layer of oxygen atoms (OI) located at z ≈ −10.4 Å, 10.4 Å were the hydroxides bonded to the surface Fe atoms (see Fig. 4c for definition of oxygen labels). The integration of these peaks produced exactly 6 oxygen atoms for the PBE96 and PBE96 + Grimme2 simulations. The second outside layer of oxygen atoms (OII) located at z ≈ −9.0 Å, 9.0 Å were the water molecules attached to the surface Fe atoms. This layer of water molecules was loosely bound to the surface, and the bond between the water O and the surface Fe^3+^ were observed to frequently break and reform during the simulations. In some cases these water molecules exchange with the more bulk-like water molecules of the next layer. The integration of these peaks produced 4.63 and 5.98 oxygen atoms for the PBE96 and PBE96 + Grimme2 simulations respectively. The first inside layer of oxygen atoms (Oi) located at z ≈ −11.9 Å, 11.9 Å were ~180° away from the OI oxygen atoms, and the second inside layer of oxygen atoms (Oii) located at z ≈ −13.0 Å, 13.0 Å were nearly ~180° away from the OII oxygen atoms.

**Fig. 6:**
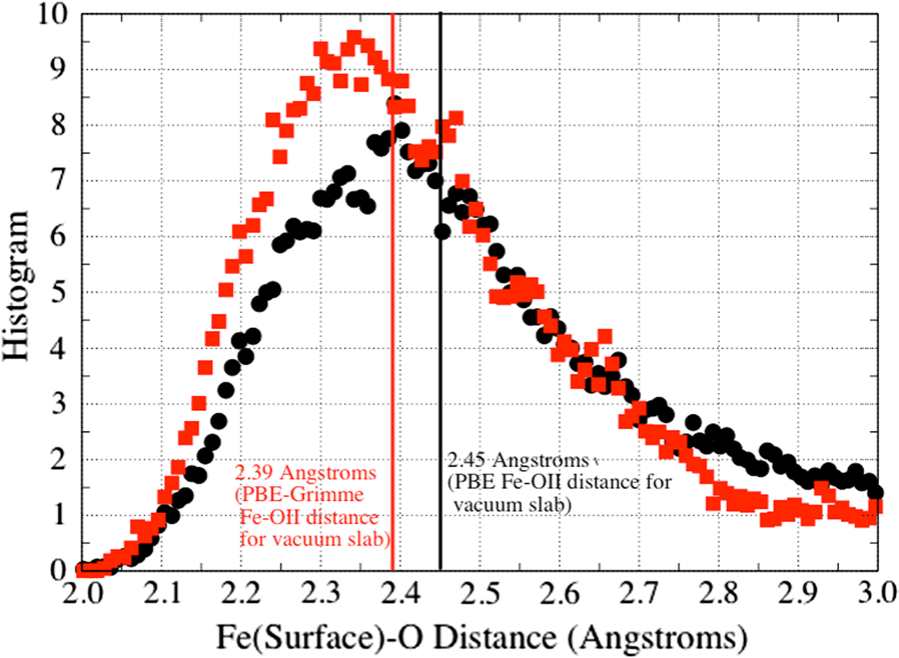
Histograms of surface Fe–OII distance from PBE96 and PBE96 + Grimme2 AIMD simulations. See Figs. 4c and 7 for definition of OII

In Table 3, the surface Fe–O bond distances and O–Fe–O bond angles for the goethite (100) surface obtained from PBE96 AIMD and PBE96 + Grimme2 AIMD along with values extracted from published experimental data are given. The maximum in the first shell peaks of Fe–O histogram shown in Fig. 6 were used for the Fe–OII distances. Except for the Fe–OII distance, the bond distances and angles for the surface slab were in in good agreement with the fitted model from CTR experiments [22]. While the Fe–OII bond distances did contract somewhat from the vacuum surface, the distances of 2.40 and 2.34 Å from the PBE96 and PBE96 + Grimme2 theories respectively, were still considerably larger than 2.15 Å in the model fitted from CTR data and the 2.21 Å micro-solvated MD calculations. The average percent difference for the rest of bond lengths with respect to the CTR values was 1.7 and 1.1% for the PBE96 and PBE96 + Grimme2 theories respectively, and for the bond angles was 2.7 and 3.1%.

**Table 3:** Comparison of surface structure parameters for the (100) surface goethite + water between PBE96 AIMD and PBE96 + Grimme2 AIMD simulations of Fe_24_O_60_H_48_ + 53 H_2_O and fitted structure from CTR experiments of Ghose et al. [22]

Surface structure parameters	PBE96 AIMD	PBE96 + Grimme2 AIMD	Exp. CTR model
R(Fe–OII)	~2.40	~2.34	2.152
σ(Fe–OII)			
R(Fe–OI)	2.10	2.11	2.097
σ(Fe–OI)	0.08	0.08	
R(Fe–Oi)	2.02	2.00	1.950
σ(Fe–Oi)	0.07	0.08	
R(Fe–Oii)	1.94	1.97	1.966
σ(Fe–Oii)	0.06	0.07	
∠OI–Fe–OII	80.2	81.1	81.25
σ ∠OI–Fe–OII	5.5	5.3	
∠OII–Fe–Oi	87.7	87.7	82.85
σ ∠OII–Fe–Oi	5.8	5.7	
∠OII–Fe–Oii	170.4	170.3	178.89
σ ∠OII–Fe–Oii	4.9	5.0	
∠OI–Fe–Oi	81.8	82.6	80.85
σ ∠OI–Fe–Oi	3.6	3.4	
∠OI–Fe–Oii	97.1	94.5	99.5
σ ∠OI–Fe–Oii	5.7	4.7	
∠OIII–Fe–Oii	97.6	97.1	96.5
σ ∠OIII–Fe–Oii	4.8	4.3	

See Fig. 4c for definitions of OI, OII, Oi, and Oii oxygens (length unit: Å, angle unit: °)

### Electronic structure of interfacial water

**Fig. 7:**
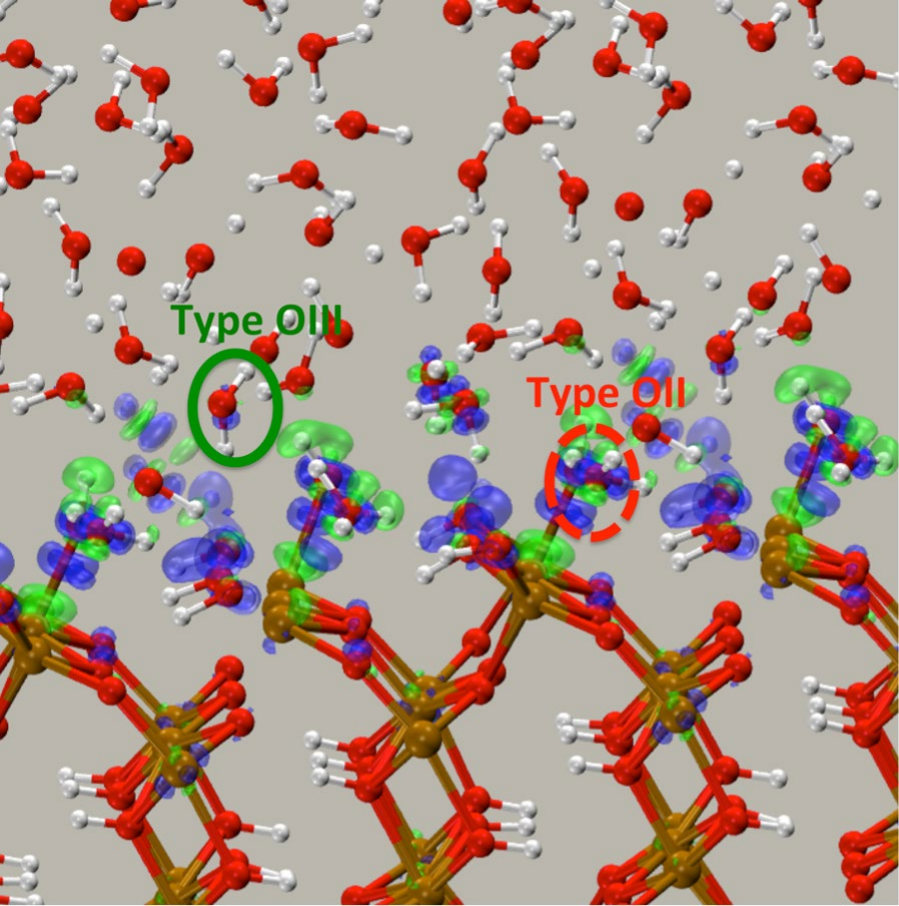
Isosurfaces of the difference in density of the full Fe_24_O_48_H_24_ + 65 H_2_O PBE96 AIMD system minus the Fe_24_O_48_H_24_ slab and the water layers (including the OII water) is shown. The surplus charge distribution is shown in the transparent *blue*: 0.005 a.u. isosurface, and the deficit charge distribution is shown in the transparent *green*: −0.005 a.u. isosurface. Configuration taken from the end of a 10 ps AIMD simulation

The proximity of the surface waters to the strongly ionic structure of the goethite surface can change the properties of solvating waters. These complex electronic structure effects are automatically included in AIMD simulations via the use of the Schrödinger equation to calculate the interatomic forces and leads to changes in the electronic structure of the surface waters. To characterize these effects we analyzed the difference in density of the full Fe_24_O_60_H_48_ + 53 H_2_O PBE96 AIMD system (or Fe_24_O_48_H_24_ + 65 H_2_O if OII water not part of slab) minus the Fe_24_O_48_H_24_ slab and the water layers (including the OII water) is shown in Fig. 7. The polarization of the water molecules in the interface layer due to the surface (and vice versa) is fairly small and localized only in the immediate vicinity of the interface, and it is concentrated in between the atoms near the OI hydroxyls and the OII waters. Note that changes in density (polarization) are also present in the solid surface region.

**Fig. 8:**
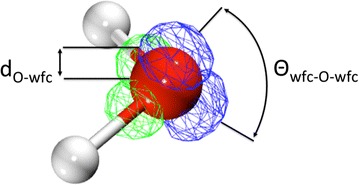
Illustration of the distance from the O center to a LPO WFC, d_O-wfc_, and the angle formed by the LPO WFCs and the O center Θ_wfc-O-wfc_. Green Wannier orbitals are bonding orbitals (BO) and the *blue* Wannier orbitals are the lone pair orbitals (LPO)

To provide a more quantifiable measure of the polarization we performed a detailed analysis of the electronic structure of the water molecules in terms of the positions of the centers of their Boys localized bond orbitals relative to the oxygen center of the water molecule (Wannier−Boys function center, WBFC, i.e. center of the Boys orbital). This representation has been shown by Lightstone et al. [65] and Bogatko et al. [66] to provide a measure of metal ion−water interactions. If there is a significant impact of the presence of the surface on the electronic structure of a water molecule, the distance from the O center to the WBFC of the lone pair orbitals (LPO) and the bond orbitals (BO) between the O and H atoms, d_O-wfc_, and the angle formed by the WBFCs and the O center Θ_wfc-O-wfc_ (see Fig. 8 for definitions) in the molecule should be altered relative to those found in bulk water. The results of this analysis, in which d_O-wfc_ and Θ_wfc-O-wfc_ are taken as averages of several independent snapshots from the Fe_24_O_60_H_48_ + 53 H_2_O PBE96 AIMD simulation, are shown in Table 4. For comparison the WBFC analysis for the homogeneous Fe^3+^−64 water, Al^3+^−64 water and the 64 water simulations from Bogatko et al. [66] are also given in the table. The analysis shows that both the spin up and spin down electrons in oxygen from type OIII water have values for d_O-wfc_ and Θ_wfc-O-wfc_ similar to those of bulk water molecules. The values for type OII water are also very close to bulk water values. However, there are small but noticeable differences. For this water the d_O-wfc_ for spin down LPO has been lengthened by ~0.02 Å, whereas the d_O-wfc_ for spin up LPO is essentially the same as bulk water. This is consistent with the weak bonding of these water molecules to the surface. Plots of the spin up and spin down LPOs (not shown) also show that the spin down LPO moves closer to the surface Fe atom whereas the spin up LPO does not. This spin dependent polarization has previously been demonstrated by Bogatko et al. [66] in the Fe^3+^−64 water system and is caused by the spin localization of the d electrons in Fe site because the spin down d orbitals for Fe^3+^ ion are empty. The effect is significantly stronger in the aqueous phase because the Fe^3+^ charge is not neutralized as it is on the surface. In the aqueous phase case the d_O-wfc_ for spin up LPO is essentially the same as for the bulk water (the spin up d orbitals for the Fe^3+^ ion are completely filled). A decrease of 8° and 3.4° has been observed for Θ_wfc-O-wfc_ spin down and spin up LPO, respectively. The BOs for the OII water have also been influenced by the polarization of LPO; the Θ_wfc-O-wfc_ for BO slightly increased and the d_O-wfc_ slightly decreased.

**Table 4:** Electronic structure for the Solvation shells represented by spin up (α), Spin down (β) Wannier Function Centers (WFC)

	OII water				OIII water				Bulk water			
	dO-wfc		Θwfc-O-wfc		dO-wfc		Θwfc-O-wfc		dO-wfc		Θwfc-O-wfc	
	BO	LPO	BO	LPO	BO	LPO	BO	LPO	BO	LPO	BO	LPO
Interface water α	0.49	0.33	104	116	0.50	0.33	103	119	0.50	0.33	103	118
Interface water β	0.49	0.35	105	111	0.50	0.33	103	119	0.50	0.33	103	118

	First hydration shell				Second hydration shell				Bulk			
	dO-wfc		Θwfc-O-wfc		dO-wfc		Θwfc-O-wfc		dO-wfc		Θwfc-O-wfc	
	BO	LPO	BO	LPO	BO	LPO	BO	LPO	BO	LPO	BO	LPO
Fe^3+^-64 H_2_O α	0.48	0.32	106	112	0.50	0.33	104	118	0.50	0.33	103	119
Fe^3+^-64 H_2_O β	0.48	0.38	110	92	0.50	0.33	104	118	0.50	0.33	103	119
Al^3+^-64 H_2_O	0.48	0.34	106	110	0.50	0.33	103	117	0.50	0.32	103	119
64 H_2_O									0.50	0.33	106	115

See Fig. 7 for definitions of OII water and OIII water (length unit: Å, angle unit: °)

These results suggest that the electronic structure of the water molecules beyond the OII water molecules has returned to that of the bulk. Even the OII interfacial waters, which are often considered part of the surface, showed very little polarization presumably because the surface has a neutral charge.

### Dynamic processes on the interface

**Fig. 9:**
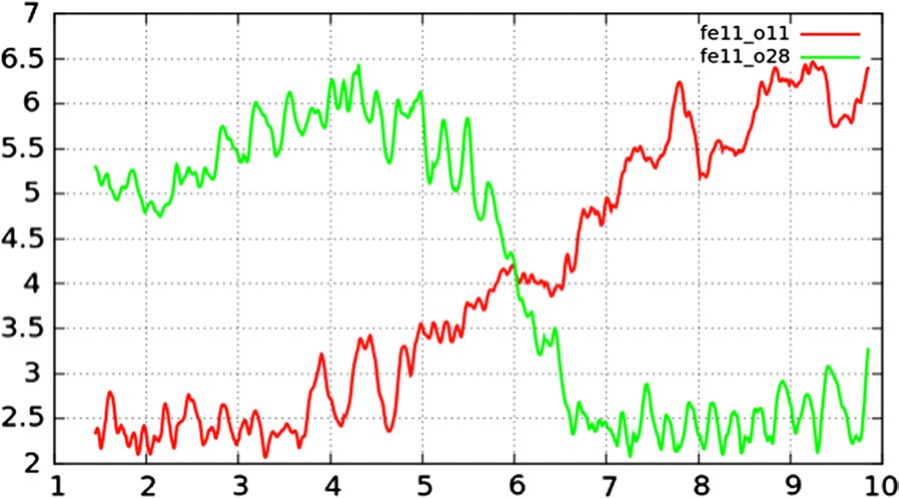
Exchange of Fe bonded OII water molecule on goethite (100) surface with bulk water

No proton transfer was observed in our AIMD simulations. While fast rates of proton exchange have been observed for some water-metal oxides interfaces [67], in particular for surfaces that are highly polarizing. Whereas for the goethite (100) surface, the lack of water dissociation at the surface is consistent with the weak interaction of the interfacial OII water to the surface, as shown in the previous section. However, by having the OII water being a weakly bound means that the barrier to it exchanging with another bulk water is likely to be low, and not surprisingly several water exchanges were found to take place between bulk water molecules and the OII water molecules (i.e. the Fe–OH_2_) on the surface in our 10 ps of simulation. We estimated the exchange rate for one Fe–OH_2_ bond on surface in the PBE96 AIMD simulation to be 0.025 exchanges/ps. However, there were not enough exchanges to calculate an accurate rate value. The ligand exchange mechanism has being widely studied for aqua ions in solutions systems [68–73]. Langford and Gray [71] proposed that substitution events may be classified into three catagories depending on the dynamic nature initiating the reaction, associative (A) (reaction initiated by one water leaving the 1st hydration shell), dissociative (D) (reaction initiated by water entering the 1st shell) and interchange (I) (simultaneous exchange of water in first hydration shell). Using this classification the exchanges we observed may be classified as dissociative (D). The process can be described as a two-step process in which the Fe–OH_2_ bond on the surface breaks first, followed by a bulk water molecule approaching the surface Fe atom. An example of a D exchange process is shown in Fig. 9. This figure shows that starting from 4 ps, the Fe–OH_2_ bond becomes unstable, and then from 5.5 to 6.5 ps, a second water molecule approaches the Fe atom while the initial water molecule slowly escapes. After 6.5 ps (or 2.5 ps in duration) the exchange process is complete.

## Conclusion

DFT optimization (including PBE96, PBE96 + Grimme2, PBE0) and AIMD simulations (PBE96 and PBE96 + Grimme2) were carried out for the anhydrous goethite (100) surface and the goethite (100) + water interface. The simulations were performed for a (3 × 2) surface slab containing 65 water molecules between the slabs (near 1 g/cm^3^) at temperature of 300 K.

The surface energies calculated from DFT PBE96 optimization of three likely fully solvated neutral surface terminations in which the Fe^3+^ cations remain in an octahedral coordination, were compared. It was found that the lowest energy surface containing an exposed surface Fe^3+^ is capped by a (weakly bound) water molecule and shares a hydroxide with a neighboring Fe^3+^ (see surface I in Fig. 2). This surface termination agrees with the assumed largest fraction surface found in fitting CTR data by Ghose et al. [22]. The other two surfaces examined, which were capped by two hydroxides, were found to be approximately 27 and 34 kJ/mol less stable per 1 × 1 surface slab (or 431 and 564 kJ/mol for the 3 × 2 surface slab). The solvation of the slabs with 53 H_2_O molecules had little effect on the relative surface energetics between the slabs. The solvated 3 × 2 slabs capped by two hydroxides (surface II and surface IID in Figs. 3 and 4) were found to have average energies that were 398 and 575 kJ/mol higher then the solvated Surface I slab (3 × 2 surface slab, Fig. 2).

For the most stable surface it was found that the water molecule formed by protonation of an unsaturated OH from the termination, and capping a surface Fe was very loosely bound (see Fig. 2). The resulting optimized or equilibrated long Fe^3+^–OH_2_ bond length does not agree with prior results obtained from fitting CTR data or from prior MD simulations [8, 27, 28, 33], which predicted Fe–OH_2_ distances only slightly longer than the bulk goethite Fe–OH distances. However, this result does agree with the prior DFT + U optimizations of Kubicki et al. [2]. Furthermore, the DFT PBE96 results were checked using hybrid PBE0 and dispersion corrected PBE96 + Grimme2 exchange correlation functionals and the results were found to change very little (weak bonding prediction upheld). These results support a terminated surface model in which the Fe^3+^ ion in the surface is coordinated by only 5 neighbors versus 6 in the bulk. Nevertheless, the surface is neutral (and not very polar). This structure was supported by bond valence (BV) theory calculations. While BV theory is essentially an empirical methodology. It has been shown to produce remarkably accurate predictions for many materials applications [60].

Full AIMD simulations of 100 goethite + water interface (Surface I cleavage) were carried out. Solvating the slabs with 53 H_2_O molecules led to a slight contraction of the distance to the nearest water from the surface (OII in Fig. 2). The average Fe–OH_2_ bond distances in the AIMD simulations that were found to be 2.40 and 2.34 Å for the PBE96 and PBE96 + Grimme2 theories respectively were still considerably larger than 2.15 Å distance seen in the model fitted from CTR data [22] and the 2.13–2.21 Å distances from prior MD calculations [27]. In summary all the DFT calculations support the long bond and weak interaction. The good agreement of the BV methodology with DFT predictions of bond length changes support the further investigation of this method for surface structures.

The polarization of the water layer due to the surface (and vice versa) is small and localized only to the immediate vicinity of the interface. Density difference plots and Wannier–Boys orbitals analysis support the classification into three types of water molecules observed in the simulations (see Fig. 7). The first is the capping water molecule (OII, Fig. 7) that is loosely bonded to the surface Fe^3+^. The second (OIII, Fig. 7) is hydrogen bonded to the surface hydroxyl, and the third type is similar to bulk water. The analysis shows that by the third layer the coordination and bonding are essentially that of bulk water. Even though the OII and OIII water molecules form ordered water layers on surface they do not bond to the surface strongly and as a result these water molecules readily exchanged with the bulk-like water molecules.

These results might suggest that other mineral surfaces may also have surface cations with only five neighbors. For instance, oxide surfaces where a surface cation is capped by a water molecule such as the (100) plane of diaspore, and the R-planes of sapphire and hematite.

## Abbreviations

AIMD: ab initio molecular dynamics
BO: bond order
CTR: crystal truncation rod
DFT: density functional theory
EXAFS: extended X-ray absorption fine structure
MD: molecular dynamics
MUSIC: multi site complexation
NWPW: NorthWest Plane-Wave module of NWChem
PBE96: Perdew, Burke and Ernzerhof 1996 exchange correlation functional
PBE0: hybrid extension of Perdew, Burke and Ernzerhof 1996 exchange correlation functional
LPO: lone pair orbital
WBFC: Wannier–Boys Function Center

## References

[1] Brown GE (1999). Metal oxide surfaces and their interactions with aqueous solutions and microbial organisms. Chem Rev.

[2] Kubicki JD, Paul KW, Sparks DL (2008). Periodic density functional theory calculations of bulk and the (010) surface of goethite. Geochem Trans.

[3] Majzlan J, Gravel KD (2003). Thermodynamics of Fe of goethite (a-FeOOH), lepidocrocite (g-FeOOF) and maghemite (g-fe2O3). Am Miner.

[4] van der Zee C (2003). Nanogoethite is the dominant reactive oxyhydroxide phase in lake and marine sediments. Geology.

[5] Hochella MF (2008). Nanominerals, mineral nanoparticles, and Earth systems. Science.

[6] Klingelhofer G (2005). Mossbauer spectroscopy on Mars: goethite in the Columbia Hills at Gusev crater. Hyperfine Interact.

[7] Villalobos M, Trotz MA, Leckie JO (2003). Variability in goethite surface site density: evidence from proton and carbonate sorption. J Colloid Interface Sci.

[8] Rustad JR, Felmy AR, Hay BP (1996). Molecular statics calculations of proton binding to goethite surfaces: a new approach to estimation of stability constants for multisite surface complexation models. Geochim Cosmochim Acta.

[9] Fendorf S (1997). Arsenate and chromate retention mechanisms on goethite. 1. Surface structure. Environ Sci Technol.

[10] Boily J-F (2000). Benzenecarboxylate surface complexation at the goethite (α-FeOOH)/water interface: I. A mechanistic description of pyromellitate surface complexes from the combined evidence of infrared spectroscopy, potentiometry, adsorption data, and surface complexation modeling. Langmuir.

[11] van Geen A, Robertson AP, Leckie JO (1994). Complexation of carbonate species at the goethite surface: implications for adsorption of metal ions in natural waters. Geochim Cosmochim Acta.

[12] Watts HD, Tribe L, Kubicki JD (2014). Arsenic adsorption onto minerals: connecting experimental observations with density functional theory calculations. Minerals.

[13] Antelo J (2005). Effects of pH and ionic strength on the adsorption of phosphate and arsenate at the goethite-water interface. J Colloid Interface Sci.

[14] Cheng T (2004). Effects of phosphate on uranium (VI) adsorption to goethite-coated sand. Environ Sci Technol.

[15] Duff M, Amrhein C (1996). Uranium (VI) adsorption on goethite and soil in carbonate solutions. Soil Sci Soc Am J.

[16] Sanchez AL, Murray JW, Sibley TH (1985). The adsorption of plutonium IV and V on goethite. Geochim Cosmochim Acta.

[17] Cockcroft J (1999) A hypertext book of crystallographic space group diagrams and tables. http://img.chem.ucl.ac.uk/sgp/mainmenu.htm

[18] Dutch S (2011) Three-dimensional space groups. http://www.uwgb.edu/dutchs/SYMMETRY/3dSpaceGrps/3dspgrp.htm

[19] Buerger MJ (1963) Elementary crystallography

[20] Rustad JR, Felmy AR, Hay BP (1996). Molecular statics calculations for iron oxide and oxyhydroxide minerals: toward a flexible model of the reactive mineral-water interface. Geochim Cosmochim Acta.

[21] Randall SR (1999). The mechanism of cadmium surface complexation on iron oxyhydroxide minerals. Geochim Cosmochim Acta.

[22] Ghose SK, Waychunas GA, Trainor TP, Eng PJ (2010). Hydrated geothite (a-FeOOH) (100) interface structure: ordered water and surface functional groups. Geochim Cosmochim Acta.

[23] Fenter P, Sturchio NC (2004). Mineral–water interfacial structures revealed by synchrotron X-ray scattering. Prog Surf Sci.

[24] Shroll RM, Straatsma TP (2003). Molecular dynamics simulations of the goethite–water interface. Mol Simul.

[25] Boily J-F (2012). Water structure and hydrogen bonding at goethite/water interfaces: implications for proton affinities. J Phys Chem C.

[26] Rustad JR, Felmy AR, Bylaska EJ (2003). Molecular simulation of the magnetite–water interface. Geochim Cosmochim Acta.

[27] de Leeuw NH, Cooper TG (2007). Surface simulation studies of the hydration of white rust Fe(OH)(2), goethite alpha-FeO(OH) and hematite alpha-Fe(2)O(3). Geochim Cosmochim Acta.

[28] Boily JF (2012). Water structure and hydrogen bonding at goethite/water interfaces: implications for proton affinities. J Phys Chem C.

[29] Kerisit S (2011). Water structure at hematite–water interfaces. Geochim Cosmochim Acta.

[30] Kohanoff J (1994). Phonon spectra from short non-thermally equilibrated molecular dynamics simulations. Comput Mater Sci.

[31] Kerisit S, Rosso KM (2006). Computer simulation of electron transfer at hematite surfaces. Geochim Cosmochim Acta.

[32] Fenter P (2013). Is the calcite–water interface understood? Direct comparisons of molecular dynamics simulations with specular X-ray reflectivity data. J Phys Chem C.

[33] Kerisit S, Ilton ES, Parker SC (2006). Molecular dynamics simulations of electrolyte solutions at the (100) goethite surface. J Phys Chem B.

[34] Hiemstra T, VanRiemsdijk WH (1996). A surface structural approach to ion adsorption: the charge distribution (CD) model. J Colloid Interface Sci.

[35] Fitts JP (2005). Second-harmonic generation and theoretical studies of protonation at the water/alpha-TiO_2_ (110) interface. Chem Phys Lett.

[36] Peacock CL, Sherman DM (2004). Vanadium(V) adsorption onto goethite (alpha-FeOOH) at pH 1.5 to 12: a surface complexation model based on ab initio molecular geometries and EXAFS spectroscopy. Geochim Cosmochim Acta.

[37] Zhou L, Xiu F, Qiu M, Xia S, Yu L (2017). The adsorption and disociation of water molecule on geothite (010)surface: A DFT approach. Appl Surf Sci.

[38] Becke AD (2014). Perspective: fifty years of density-functional theory in chemical physics. J Chem Phys.

[39] Brown ID (1987). Recent developments in the bond valence model of inorganic bonding. Phys Chem Miner.

[40] Car R, Parrinello M (1985). Unified approach for molecular-dynamics and density-functional theory. Phys Rev Lett.

[41] Marx D, Hutter J (2012). Ab initio molecular dynamics: basic theory and advanced methods.

[42] Bylaska E et al. (2011) Large‐scale plane‐wave‐based density functional theory: formalism, parallelization, and applications. In: Computational methods for large systems: electronic structure approaches for biotechnology and nanotechnology, pp 77–116

[43] Valiev M, Bylaska EJ, Govind N, Kowalski K, Straatsma TP, Van Dam HJ, Wang D, Nieplocha J, Apra E, Windus TL, De Jong WA (2010) NWChem: a comprehensive and scalable open-source solution for large scale molecular simulations. Comput Phys Commun 181(9):1477–1489

[44] Perdew JP, Burke K, Ernzerhof M (1997). Generalized gradient approximation made simple (vol 77, pg 3865, 1996). Phys Rev Lett.

[45] Grimme S (2010). A consistent and accurate ab initio parametrization of density functional dispersion correction (DFT-D) for the 94 elements H-Pu. J Chem Phys.

[46] Gillan MJ, Alfe D, Michaelides A (2016). Perspective: how good is DFT for water?. J Chem Phys.

[47] Adamo C, Barone V (1999). Toward reliable density functional methods without adjustable parameters: the PBE0 model. J Chem Phys.

[48] Bogatko S (2013). The aqueous Ca^2+^ system, in comparison with Zn^2+^, Fe^3+^, and Al^3+^: an ab initio molecular dynamics study. Chem Eur J.

[49] Hamann DR (1989). Generalized norm-conserving pseudopotentials. Phys Rev B.

[50] Hamann DR, Schluter M, Chiang C (1979). Norm-conserving pseudopotentials. Phys Rev Lett.

[51] Troullier N, Martins JL (1991). Efficient pseudopotentials for plane-wave calculations. Phys Rev B.

[52] Bylander DM, Kleinman L (1984). Outer-core electron and valence electron pseudopotential. Phys Rev B.

[53] Rochester CH, Topham SA (1979). Infrared study of surface hydroxyl-groups on goethite. J Chem Soc Faraday Trans.

[54] Ying C, Bylaska E, Weare J, Kubicki J (2016). Chapter 4: 1st principle estimation of geochemically important transition metal oxide properties: structure and dynamics of the bulk, surface and mineral/aqueous fluid interface. Molecular modeling of geochemical reactions: an introduction.

[55] Rosso KM, Rustad JR (2001). Structures and energies of AlOOH and FeOOH polymorphs from plane wave pseudopotential calculations. Am Miner.

[56] Tunega D (2012). Theoretical study of properties of goethite (α-FeOOH) at ambient and high-pressure conditions. J Phys Chem C.

[57] Nose S (1984). A unified formulation of the constant temperature molecular-dynamics methods. J Chem Phys.

[58] Hoover WG (1985). Canonical dynamics—equilibrium phase-space distributions. Phys Rev A.

[59] Blochl PE, Parrinello M (1992). Adiabaticity in 1st-principles molecular-dynamics. Phys Rev B.

[60] Brown ID, Altermatt D (1985). Bond-valence parameters obtained from a systematic analysis of the inorganic crystal-structure database. Acta Crystallogr Sect B Struct Sci.

[61] Donnay G, Allman R (1970) How to recognize O^2−^, OH^−^, and H_2_O in crystal structures determined by X-rays. Am Mineral 55(5–6):1003

[62] Brown ID (2009). Recent developments in the methods and applications of the bond valence model. Chem Rev.

[63] Pauling L (1929). The principles determining the structure of complex ionic crystals. J Am Chem Soc.

[64] Bergerhoff G (1983). The inorganic crystal-structure data-base. J Chem Inf Comput Sci.

[65] Lightstone FC (2005). A first-principles molecular dynamics study of calcium in water. ChemPhysChem.

[66] Bogatko SA, Bylaska EJ, Weare JH (2010). First principles simulation of the bonding, vibrational, and electronic properties of the hydration shells of the high-spin Fe^3+^ ion in aqueous solutions. J Phys Chem A.

[67] Kumar N (2011). Faster proton transfer dynamics of water on SnO_2_ compared to TiO_2_. J Chem Phys.

[68] Bogatko S (2013). The aqueous Ca^2+^ system, in comparison with Zn^2+^, Fe^3+^, and Al^3+^: an ab initio molecular dynamics study. Chem Eur J.

[69] Bylaska EJ (2007). Structure and dynamics of the hydration shells of the Al^3+^ ion. J Chem Phys.

[70] Richens DT (2005). Ligand substitution reactions at inorganic centers. Chem Rev.

[71] Langford CH, Gray HB (1965). Ligand substitution processes.

[72] Nichols P (2008). Equatorial and apical solvent shells of the UO22+ ion. J Chem Phys.

[73] Atta-Fynn R (2011). Hydration shell structure and dynamics of curium (III) in aqueous solution: first principles and empirical studies. J Phys Chem A.

